# Automated Analysis of Risk Factors for Postictal Generalized EEG Suppression

**DOI:** 10.3389/fneur.2021.669517

**Published:** 2021-05-11

**Authors:** Xiuhe Zhao, Laura Vilella, Liang Zhu, M. R. Sandhya Rani, Johnson P. Hampson, Jaison Hampson, Norma J. Hupp, Rup K. Sainju, Daniel Friedman, Maromi Nei, Catherine Scott, Luke Allen, Brian K. Gehlbach, Stephan Schuele, Ronald M. Harper, Beate Diehl, Lisa M. Bateman, Orrin Devinsky, George B. Richerson, Guo-Qiang Zhang, Samden D. Lhatoo, Nuria Lacuey

**Affiliations:** ^1^Department of Neurology, Qilu Hospital of Shandong University, Jinan, China; ^2^National Institute of Neurological Disorders and Stroke (NINDS) Center for Sudden Unexpected Death in Epilepsy (SUDEP) Research, McGovern Medical School, University of Texas Health Science Center at Houston, Houston, TX, United States; ^3^Department of Neurology, McGovern Medical School, University of Texas Health Science Center at Houston, Houston, TX, United States; ^4^Biostatistics and Epidemiology Research Design Core, Division of Clinical and Translational Sciences, McGovern Medical School, University of Texas Health Science Center at Houston, Houston, TX, United States; ^5^Department of Neurology, University of Iowa Carver College of Medicine, Iowa City, IA, United States; ^6^New York University (NYU) Grossman School of Medicine, New York, NY, United States; ^7^Sidney Kimmel Medical College, Thomas Jefferson University, Philadelphia, PA, United States; ^8^Department of Neurology, Institute of Neurology, University College London, London, United Kingdom; ^9^Feinberg School of Medicine, Northwestern University, Chicago, IL, United States; ^10^Department of Neurobiology and the Brain Research Institute, University of California, Los Angeles, Los Angeles, CA, United States; ^11^Department of Neurology, Cedars-Sinai Medical Center, Los Angeles, CA, United States

**Keywords:** PGES, generalized convulsive seizure, epilepsy, SUDEP, mortality, post-ictal generalized EEG suppression, sudden unexpected death in epilepsy

## Abstract

**Rationale:** Currently, there is some ambiguity over the role of postictal generalized electro-encephalographic suppression (PGES) as a biomarker in sudden unexpected death in epilepsy (SUDEP). Visual analysis of PGES, known to be subjective, may account for this. In this study, we set out to perform an analysis of PGES presence and duration using a validated signal processing tool, specifically to examine the association between PGES and seizure features previously reported to be associated with visually analyzed PGES.

**Methods:** This is a prospective, multicenter epilepsy monitoring study of autonomic and breathing biomarkers of SUDEP in adult patients with intractable epilepsy. We studied videoelectroencephalogram (vEEG) recordings of generalized convulsive seizures (GCS) in a cohort of patients in whom respiratory and vEEG recording were carried out during the evaluation in the epilepsy monitoring unit. A validated automated EEG suppression detection tool was used to determine presence and duration of PGES.

**Results:** We studied 148 GCS in 87 patients. PGES occurred in 106/148 (71.6%) seizures in 70/87 (80.5%) of patients. PGES mean duration was 38.7 ± 23.7 (37; 1–169) seconds. Presence of tonic phase during GCS, including decerebration, decortication and hemi-decerebration, were 8.29 (CI 2.6–26.39, *p* = 0.0003), 7.17 (CI 1.29–39.76, *p* = 0.02), and 4.77 (CI 1.25–18.20, *p* = 0.02) times more likely to have PGES, respectively. In addition, presence of decerebration (*p* = 0.004) and decortication (*p* = 0.02), older age (*p* = 0.009), and hypoxemia duration (*p* = 0.03) were associated with longer PGES durations.

**Conclusions:** In this study, we confirmed observations made with visual analysis, that presence of tonic phase during GCS, longer hypoxemia, and older age are reliably associated with PGES. We found that of the different types of tonic phase posturing, decerebration has the strongest association with PGES, followed by decortication, followed by hemi-decerebration. This suggests that these factors are likely indicative of seizure severity and may or may not be associated with SUDEP. An automated signal processing tool enables objective metrics, and may resolve apparent ambiguities in the role of PGES in SUDEP and seizure severity studies.

## Introduction

Sudden unexpected death in epilepsy (SUDEP) incidence ranges from 1.1 to 5.9 per 1,000 patient-years in epilepsy clinic populations to 6.3–9.3 per 1,000 patients with intractable epilepsy, posing a significant public health problem (1). Risk factors that increase risk include ≥3 generalized convulsive seizure (GCS) per year, younger age of onset of epilepsy and longer duration of epilepsy (2–4). In a case-control study (5), duration of postictal generalized electroencephalographic suppression (PGES) was significantly longer in patients who later died of SUDEP; duration >50 s was significantly associated with a higher risk of SUDEP (5). All patients in the Mortality in Epilepsy Monitoring Units Study (MORTEMUS) had PGES in the agonal seizure (6). However, a case control study of patients who later died of SUDEP did not find PGES predictive (7). Although several observations have been made on factors associated with PGES, seizure severity, and potential SUDEP risk, almost all of have been made with visual analysis of PGES, which we now know to be subjective and perhaps unreliable. This may explain apparent ambiguity over the role of PGES in SUDEP (8–10). In this study, we set out to perform an analysis of PGES presence and duration using a validated signal processing tool (11), specifically to examine the association between PGES and seizure features previously reported to be associated with visually analyzed PGES.

## Methods

All patients were prospectively consented participants in the National Institute of Neurological Disorders and Stroke (NINDS) Center for SUDEP Research's (CSR) Autonomic and Imaging Biomarkers of SUDEP multicenter project (U01-NS090407) and its preliminary phase, the Prevention and Risk Identification of SUDEP Mortality (PRISM) project (P20NS076965). We studied video-electroencephalogram (vEEG) recordings of GCS in a cohort of patients who underwent long-term monitoring in the epilepsy monitoring unit (EMU). Generalized convulsive seizures (GCS) included generalized tonic-clonic seizures, focal to bilateral tonic-clonic seizures, and focal-onset motor bilateral clonic seizures (12).

The cohort included a total of 87 patients who had 148 GCS, in whom inductance plethysmography (abdominal and thoracic belts) and vEEG recording were carried out during the evaluation in EMU. EEGs were recorded on Nihon Kohden EEG acquisition software with a 1,000 Hz sampling rate using conventional bipolar and common average referenced 10–20 montages. Chest and abdominal excursions were recorded using inductance plethysmography (Ambu, Ballerup, Denmark). Peripheral capillary oxygen saturation (SpO_2_) and heart rate (HR) were monitored using pulse oximetry (NellcorOxiMax N-600x, Covidien, Minneapolis, MN, USA). The cardiopulmonary data were time-synchronized with the EEG data.

A validated automated EEG suppression detection tool (11) was used to determine presence and duration of PGES and supplemented by two-experienced clinical epileptologists (SL and NL) who reviewed the EEG records with differences resolved by consensus when the tool gave no solution.

### Collection of Variables

The following data were collected and analyzed: Age on admission and at epilepsy onset, gender, body mass index (BMI), duration of epilepsy, previous epilepsy surgery and number of GCS in the preceding 12 months (classified as ≥3 and <3), seizure semiological presentation and duration, body position at seizure end before nursing intervention (supine, prone, lateral, sitting, or undetermined), state prior to seizure onset (awake/sleep and sleep state [N1, N2, N3, and R]).

Tonic phase was defined as tonicity in both arms, further classified into decerebration (both upper limbs straight symmetric), decortication (both upper limbs flexed symmetrically) or hemi-decerebration (tonic extension of one arm with flexion of contralateral arm without progression to decortication or decerebration). Postictal generalized electroencephalographic suppression (PGES), postictal burst suppression and EEG recovery in the post-convulsive period were identified. The EEG signal-processing algorithm was trained to recognize PGES as in previous studies (5, 13): an immediate postictal (within 30 s after GCS) generalized absence of electroencephalographic activity >10 μV in amplitude, lasting at minimum 1 s. Duration of PGES referred to the time interval from PGES onset to the appearance of burst suppression or continuous slowing. Burst Suppression (BS) was defined as generalized, intermittent slowing during the post-convulsive period, alternating with PGES. Combined PGES and burst suppression made up the EEG recovery duration.

Localization of the putative epileptogenic zone was defined based on clinical history, seizure semiology, neuroimaging, and EEG. High-resolution magnetic resonance imaging (MRI) or computed tomography (CT) results were collected (Table 1). The number of antiepileptic drugs (AED) on admission and changes of AED before the recorded GCS (tapering, withdrawal, increase or no change) were recorded. Early nursing intervention was defined as oxygen administration or oral suctioning applied during the seizure or within 5 s of seizure termination. Respiratory data included peripheral arterial oxygen saturation (SpO_2_) at preictal baseline, GCS onset, SpO_2_ nadir, duration of hypoxemia (<90%, from GCS onset to 3 min postictal), and presence of central apnea (pre-GCS or post-GCS) were collected using respiratory inductance plethysmography and finger pulse oximetry. Central apnea was defined as ≥1 missed breaths without any other explanation (i.e., speech, movement, or intervention). In some cases whose respiratory data were missing, review of the video to assess respiratory effort helped in differentiating obstructive from central apnea.

**Table 1:** Seizure-specific demographic, clinical characteristics and MRI neuroimaging details.

		**Total** **(*n* = 148)**	**PGES** **(*n* = 106)**	**No PGES** **(*n* = 42)**
**Demographics**		***N***	***N***	***N***
Gender	Male	67	49	18
	Female	81	57	24
Age at admission (mean), years		37.37	38.12	36.02
Age at epilepsy onset (mean), years		19.71	19.56	16.64
BMI		28.99	29.62	27.38
**History**				
Duration of epilepsy (mean), years		17.54	17.58	16.69
Previous epilepsy surgery		16	8	8
Frequency of GCS in the previous year	<3	33	22	11
	≥3	100	72	28
**Epileptogenic zone**				
Temporal		37	34	3
Frontal		23	15	8
Parietal		1	1	0
Occipital		1	1	0
Multifocal		23	13	10
Generalized		28	23	5
Lateralized		20	11	9
Bitemporal		12	5	7
Unknown		3	3	0
**Lateralization of seizure onset**				
Left		49	36	13
Right		33	26	7
Generalized		28	23	5
Bilateral		35	18	17
**State at seizure onset**	Awake	72	53	19
	Sleep	73	53	20
**Sleep stage at seizure onset**	R	1	1	0
	N1	22	14	8
	N2	49	38	11
	N3	1	0	1
**Neuroimaging findings on MRI**				
Negative		90	59	13
Positive				
Tumor		10	7	3
Encephalomalacia		8	7	1
Hippocampal asymmetry		6	5	1
Vascular malformation		4	3	2
Temporal horn asymmetry		4	2	2
Mesial temporal sclerosis		2	0	2
Malformation of cortical development		6	6	0
Temporal horn asymmetry		4	2	2
Unavailable		18	17	1
**AED**				
Number of AED on admission (mean)		2	1.88	2.31
	No AED	1	1	0
	One	38	33	5
	Two	75	54	21
	Three	28	14	14
	Four	6	4	2
AED changes before GTCS				
	Tapering	69	40	29
	Withdrawal	70	61	9
	Increase	2	1	1
	No change	7	4	3
**Early nursing interventions**				
	O_2_ administration	84	59	25
	Oral suction	62	49	13

*EEG, electroencephalogram; PGES, postictal generalized EEG suppression; GCS, generalized clonic seizure; BMI, body mass index; AED, antiepileptic drugs; R, REM*.

### Statistical Analysis

Descriptive statistics were provided for seizure-specific demographic and clinical variables (Table 1). Generalized estimating equation (GEE) method was used to model the presence of PGES or PGES duration on different demographic and clinical variables. Variables with a *p* < 0.1 were employed in the multivariate models. For the binary variable presence of PGES, a binary distribution and a logit link were specified in GEE and odds ratios, 95% confidence intervals, and *p*-values were reported (**Table 3**). For the continuous variable PGES duration, covariate coefficient estimates, standard error, and corresponding *p*-values from GEE method were provided (**Table 4**). *P* < 0.05 in the final models were considered significant. All analyses were performed in SAS 9.4 (Cary, NC).

## Results

Data from 148 GCS in 87 patients were collected. These patients were previously reported in a paper studying breathing changes as biomarkers of SUDEP (13). Details of seizure-specific demographics, clinical characteristics and MRI neuroimaging details are presented in Table 1. Seizure-specific electroclinical seizure features and cardiorespiratory data are shown in Table 2.

**Table 2:** Seizure-specific electroclinical features and cardiorespiratory data of the patients.

		**Total** **(*n* = 148)**	**PGES** **(*n* = 106)**	**No PGES** **(*n* = 42)**
**Seizure semiology**				
Duration of generalized seizure (mean), s		49.22	47.35	51.88
Duration of tonic phase (mean), s		8.74	8.38	10.2
Duration of clonic phase (mean), s		34.97	33.12	38.12
Tonic phase semiology				
	Hemi-decerebration	13	13	0
	Decerebration	66	59	7
	Decortication	29	21	8
	No tonic	40	13	27
**Body position (at seizure end)**				
	Supine	98	73	25
	Prone	4	1	3
	Lateral	41	27	14
	Sitting	1	1	0
	Undermined	4	4	0
**EEG**				
PGES duration (mean), s		38.73		
Burst suppression		62	56	6
EEG recovery (mean), s		83.44	82.07	92.14
**Respiratory data**				
SpO_2_ at baseline (mean), %		95.46	94.35	95.55
SpO_2_ at GCS onset (mean),%		92.55	93.1	90.68
SpO_2_ nadir (mean), %		59.64	57.07	67.22
Duration of hypoxemia (mean), s		130.82	132.34	126.33
Central apnea				
	Ictal	49	36	13
	Postictal	31	27	4

*Values are given as numbers of GCS unless otherwise indicated.*

*EEG, electroencephalogram; PGES, postictal generalized EEG suppression; GCS, generalized clonic seizure; SpO_2_, peripheral capillary oxygen saturation*.

Mean age on admission was 37.4 ± 14.1 (34; 18–77) years and at epilepsy onset was 19.7 ± 16.06 (16–67) years. Epilepsy duration was 17.5 ± 12.2 (15; 0.08–45) years, with 65% more than 10 years. Mean body mass index was 28.7 ± 6.6 kg/m^2^ (28.1; 15.1–45.8 kg/m^2^). Twenty-six patients had <3 GCS in the last year and 52 had ≥3. Frequency of GCS was unknown in nine patients. Sixteen (10.8%) had previous epilepsy surgery. Ninety (60.8%) of the seizures were recorded from 50 patients who had negative neuroimaging (details seen in Table 1).

Seizures were of temporal onset in 37(25%), generalized in 28(18.9%), frontal in 23(15.5%), multifocal in 23(15.5%), lateralized but not localized in 20 (13.5%), bitemporal in 12(8.1%), occipital in one (0.7%), and parietal in one (0.7%). Seizure onset could not be localized or lateralized in the remaining three seizures. Seizure onset was left in 49 (33.1%), right in 33 (22.3%), bilateral in 35 (23.6%), and generalized in 28 (18.9%). The remaining three were undetermined. At the time of the recorded seizure, patients were on three or more AEDs in 23% seizures, on two in 50.7%, on one in 25.7%, and 0.7% on no AEDs. Oral suctioning was used in 62/148 (41.89%) seizures and oxygen administration on 84/148 (56.76%).

Tonic semiology was seen in 108/148 (72.9%) seizures, with decerebration in 66/148 (44.6%), decortication in 29/148 (19.6%), and hemi-decerebration in 13/148 (8.8%) (Table 2) The mean duration of tonic phase was 8.7 (8.50, 1–23) seconds (s). The mean duration of the clonic phase was 34.9 (32–110) seconds and of the entire GCS, 49.22 (48, 5–126) seconds, Table 2.

Seventy-two seizures occurred during wakefulness, 73 during sleep, and the remaining three (in one patient) during postictal stupor due to a seizure cluster. Out of 72 seizures that occurred during wakefulness, 53 had PGES. Out of 73 seizures that occurred during sleep, 52 had PGES. The duration of PGES in seizures arising from awake state was 41.52 (2–169) seconds, and 35.83 (1–119) seconds when asleep. Supine posture was seen after 98/148 (66%) seizures with mean PGES duration of 38.89 (2–169). Lateral posture was seen after 41/148 (28%) seizures with mean PGES duration of 31.25 (23–36) seconds. Prone posture was seen in 4/148 (28%) with mean PGES duration of 38.00 (28–42) seconds. Sitting posture was grouped with supine due to the small number (one patient), and undetermined was excluded from analysis (Table 2).

Mean baseline SpO_2_ was 95.46 ± 2.35 (96; 89–100)%. The SpO_2_ nadir mean was 59.64 ± 14.10 (60; 22–87)%. In 33% (49/148) seizures, central apnea occurred before GCS (ictal central apnea, ICA) and in 21% (31/148), apnea occurred postictally (post-convulsive central apnea, PCCA) (Table 2).

### PGES (Postictal Generalized EEG Suppression)

PGES occurred in 106/148 (71.6%) seizures and 70/87 (80.5%) patients (57 female). Mean duration was 38.7 ± 23.7 (37; 1–169) seconds (s). PGES duration ≥50 s occurred in 18% (19/106). EEG recovery duration was 83.4 + 119.2 (54; 1–1,091) seconds. In univariate analysis, we found that older age was associated with longer PGES duration (*p* = 0.03) and higher BMI was associated with the presence of PGES (*p* = 0.04). Gender, age at epilepsy onset, epilepsy duration, previous epilepsy surgery, and the number of GCS within 12 months prior to admission were not associated with PGES. Epileptogenic zone and imaging abnormalities were not associated with occurrence or duration of PGES. During EMU monitoring, most patients had changes in AEDs (tapering: 46.6%, withdrawal: 47.3%).The number of AEDs at admission or changes before recorded GCS were not correlated with PGES presence or duration. Similarly, presence or duration of PGES were not associated with early nursing intervention (oxygen administration or oral suctioning).

In univariate analysis, presence of decerebration (*p* < 0.01), decortication (*p* = 0.01), and hemidecerebration (*p* < 0.0001) during tonic phase were all associated with PGES presence. In addition, decerebration was associated with longer PGES duration (*p* = 0.009). Duration of GCS, tonic or clonic phase were not associated with PGES incidence (*p* = 0.39; *p* = 0.54; *p* = 0.26, respectively) or PGES duration (*p* = 0.46; *p* = 0.37; *p* = 0.35). Sleep (*p* = 0.1) and lateral posture (*p* = 0.04) were inversely correlated to duration of PGES. Severe hypoxemia (SpO_2_ <75%) was associated with PGES presence (*p* = 0.007) and duration (*p* = 0.003); hypoxemia duration in turn was associated with PGES duration (*p* = 0.03). For central apnea, there was no association with occurrence of ICA or PCCA and PGES.

In multivariate analysis, presence of decerebration, decortication and hemi-decerebration during the tonic phase, were 8.29 (CI 2.6–26.39, *p* < 0.001), 7.17 (CI 1.29–39.76, *p* = 0.02), and 4.77 (CI 1.25–18.20, *p* = 0.02) times more likely to have PGES, respectively, compared to absence of tonic phase (Table 3). In addition, decerebration presence (*p* = 0.004), decortication presence (*p* = 0.02), older age (*p* = 0.009), and hypoxemia duration (*p* = 0.03) were associated with longer PGES durations. Sleep state (as opposed to awake) was associated with shorter PGES duration (*p* = 0.001; Tables 3, 4).

**Table 3:** Multivariate analysis of electroclinical seizure features and cardiorespiratory data with PGES presence.

		**OR**	**Confidence interval**	***P-*value**
Tonic phase semiology	Decerebration	8.29	2.60–26.39	**0.0003**
	Decortication	4.77	1.25–18.20	**0.02**
	Hemi-decerebration	7.17	1.29–39.76	**0.02**
	No tonic	–	–	–
Lateralization of seizure onset	Right	0.90	0.23–3.51	0.88
	Generalized	0.93	0.17–5.21	0.93
	Bilateral	0.40	0.12–1.32	0.13
	Left	–	–	–
Post-convulsive central apnea (PCCA)	Yes	1.32	0.44-3.98	0.62
	No	–	–	–
BMI		1.04	0.97–1.11	0.24

*Bold value indicates the statistically significant (p < 0.05)*.

**Table 4:** Multivariate analysis of electroclinical seizure features and cardiorespiratory data with PGES duration.

		**Estimate**	**SE**	***p-*value**
Tonic phase semiology	Decerebration	34.97	12.15	**0.004**
	Decortication	23.85	10.41	**0.02**
	Hemi-decerebration	17.29	12.70	0.17
	No tonic	–	–	–
Position at GCS end	Supine/Sitting	12.21	5.92	**0.039**
	Lateral	–	–	–
Age		0.77	0.29	**0.009**
GCS Onset O_2_		−0.42	0.34	0.22
Hypoxemia duration		0.10	0.04	**0.03**
State	Sleep	−13.54	4.21	**0.001**
	Awake	–	–	–
BMI		0.07	0.25	0.77

*GCS, generalized convulsive seizure; BMI, body mass index. Bold value indicates the statistically significant (p < 0.05)*.

## Discussions

We confirmed, using an objective method for analysis of PGES, that several variables indicated by visual analysis were indeed associated with this phenomenon. PGES is a common phenomenon following GCS, with an incidence of 72%, and has an average duration of 38.7 s. PGES occurrence is associated with presence of a tonic phase during GCS and presence of severe hypoxemia. In addition, longer durations of tonic phase and hypoxemia, older age and postictal supine and sitting position (compared to lateral) are associated with longer PGES duration.

Reports of PGES incidences are variable, ranging from 29 to 74% after GCS (5, 7–10, 14–17). Variability may be due to observer subjectivity, heterogeneous patient populations, and different criteria for diagnosis (7, 18). Differences in visual analysis may be significant; we now know this to be subjective and perhaps unreliable. Our results (72%) indicate PGES to be reliably common. Average PGES duration in our study was 38.7 s. This is roughly concordant with previous studies where PGES duration ranged from 33 to 53 s (8, 10, 17), with a median of 28 s (8). Only 18% of GCS had PGES duration longer than >50 s, suggesting prolonged obtundation is usually infrequent. Prolonged PGES has been suggested as a SUDEP risk factor (5) and associated with more severe postictal coma (17), although other studies have not confirmed this relationship (7, 8). One study found that patients who died of SUDEP had significantly shorter PGES duration compared to living controls (19). In our prospective cohort, two near SUDEP cases occurred (13), with PGES lasting 169 and 75 s, respectively, although no conclusions can be drawn from this observation alone. Analysis of the overall CSR study (of which this report is a part) is currently under way.

We found that presence of tonic phase is associated with both appearance and longer duration of PGES confirming studies based on visual analysis on tonic phase presence (9, 10, 16, 17, 20) and tonic phase duration (9). Different types of tonic phase posturing had varying impact, such that decerebration had the strongest association, followed by decortication, followed by hemi-decerebration. We found that lateral posture (compared to supine) at seizure end is inversely correlated with PGES duration, suggesting briefer obtundation. Although SUDEP cases are more likely to be found in a prone position (21, 22), we could not assess possible associations due to the limited number of prone individuals [4/148 (2.7%)] in our cohort. Sleep state at seizure onset was inversely associated with PGES duration. This is in contrast to other studies that have noted direct association between sleep and increased PGES (8, 10, 23), although others have not (19, 20, 24). Deep sleep before seizure onset, including REM (R) and N3 sleep only occurred in one patient each, and hence the relationship between sleep state and PGES is unresolved here.

In our study, we found that older age at admission was correlated with longer PGES. This is consistent with previous studies (25, 26), but not with others (8–10). We did not find any association between temporal lobe seizure onsets and occurrence of PGES, concordant with some studies (8, 10), but not with others (7, 17) Consistent with all previous studies, we found no association between PGES and GCS frequency, total duration of GCS, gender, epilepsy duration or presence of MRI abnormalities (8–10, 15, 24, 27). AED reduction has been said to increase risk of PGES five-fold (8). Our results suggest that AED reduction did not influence PGES, consistent with a previous study (16), although this lack or relationship may reflect a very small number who did not have medications decreased. Early periictal nursing intervention (14) and O_2_ administration (10) are reportedly been associated with reduced PGES duration. Other studies found intervention, including early O_2_ mask administration to have no effect (19, 20, 28), consistent with our current findings. However, hypoxemia duration was associated with PGES duration, reflecting the finding that O_2_ administration was local EMU protocol driven (regardless of hypoxemia extent) rather than governed by the extent of hypoxemia encountered. This interpretation is in line with ictally triggered severe hypoxemia and hypercapnia previously observed with PGES (14, 15, 29, 30).

One of the main limitations of our study is that the results are largely confirmatory of prior results achieved by visual analysis, and lacks of novel findings. However, the reproducibility of the results may be an indicator that the automated PGES tool is reliable for research studies in order to eliminate the inter-rater variability in PGES assessment in a time-sparing manner.

## Conclusions

In this study, with a custom signal-processing tool for PGES analysis, we confirmed observations made with visual analysis, that presence of a tonic phase during GCS, longer hypoxemia, older age and postictal supine or sitting position (compared to lateral) are reliably associated with PGES. The findings suggest that these factors are likely indicative of seizure severity and may or may not be associated with SUDEP. We found that of the different types of tonic phase posturing, decerebration has the strongest association with PGES, followed by decortication, followed by hemi-decerebration. Part of the variability in the literature could result from heterogeneous patient populations, different research methodologies, anti-epileptic medications, or seizure frequency. However, use of visual analysis of PGES detection and quantification, likely plays a part. An automated signal-processing tool enables objective metrics, and may resolve apparent ambiguities in the role of PGES in SUDEP and seizure severity studies.

## Data Availability Statement

The raw data supporting the conclusions of this article will be made available by the authors, without undue reservation.

## Ethics Statement

The studies involving human participants were reviewed and approved by the studies involving human participants were reviewed and approved by Case Western Reserve University and University Hospitals, as part of NIH/NINDS Center for SUDEP Research study (U01 NS090405, U01 NS090406, and U01 NS090407). Written informed consent to participate in this study was provided by the participants' legal guardian/next of kin. The patients/participants provided their written informed consent to participate in this study.

## Author Contributions

XZ and LV were major role in data acquisition, data analysis and interpretation, and drafted manuscript for intellectual content. NL designed and conceptualized the study, interpretation of the data, and drafted manuscript for intellectual content. SL designed and conceptualized the study, and revised the manuscript for intellectual content. RS, DF, MN, BG, SS, RH, BD, LB, OD, GR, and G-QZ revised the manuscript for intellectual content. JoH, JaH, MR, NH, CS, and LA were involved in the data acquisition and management. LZ was involved in the statistical analysis. All authors contributed to the article and approved the submitted version.

## Conflict of Interest

OD receives grant support from NINDS, NIMH, MURI, CDC, and NSF. He has equity and/or compensation from the following companies: Tilray, Receptor Life Sciences, Qstate Biosciences, Tevard Biosciences, Script Biosciences, Regel Biosciences, Empatica, Papa & Barkley, Rettco, Silver Spike, and California Cannabis Enterprises (CCE). The remaining authors declare that the research was conducted in the absence of any commercial or financial relationships that could be construed as a potential conflict of interest.
